# Rab32 facilitates Schwann cell pyroptosis in rats following peripheral nerve injury by elevating ROS levels

**DOI:** 10.1186/s12967-024-04999-x

**Published:** 2024-02-22

**Authors:** Jiayi Wang, Pin Chen, Guanjie Han, Yongjie Zhou, Xingdong Xiang, Mengxuan Bian, Lei Huang, Xiang Wang, Binfeng He, Shunyi Lu

**Affiliations:** 1 Department of Orthopedic Surgery, Zhongshan Hospital, Fudan University, Shanghai, China; 2 Department of Neurosurgery, Zhongshan Hospital, Fudan University, Shanghai, China; 3 Department of Interventional Radiology, Zhongshan Hospital, Fudan University, Shanghai, China; 4 Department of Rehabilitation, Zhongshan Hospital, Fudan University, Shanghai, China; 5 Department of Cardiology, The First Affiliated Hospital of Nanchang University, Nanchang, Jiangxi 330006 China; 6 Department of Cardiology, Zhongshan Hospital, Fudan University, Shanghai, China; 7 Department of Pulmonary and Critical Care Medicine, Zhongshan Hospital, Fudan University, Shanghai, China; 8 Department of Genel Practice, Xinqiao Hospital, Third Military Medical University, Chongqing, China; 9 Department of Orthopedic Surgery, The First Affiliated Hospital of Soochow University, Suzhou, Jiangsu China

**Keywords:** Peripheral nerve regeneration, Pyroptosis, Schwann cells, Rab32

## Abstract

**Background:**

Peripheral nerve injury (PNI) is commonly observed in clinical practice, yet the underlying mechanisms remain unclear. This study investigated the correlation between the expression of a Ras-related protein Rab32 and pyroptosis in rats following PNI, and potential mechanisms have been explored by which Rab32 may influence Schwann cells pyroptosis and ultimately peripheral nerve regeneration (PNR) through the regulation of Reactive oxygen species (ROS) levels.

**Methods:**

The authors investigated the induction of Schwann cell pyroptosis and the elevated expression of Rab32 in a rat model of PNI. In vitro experiments revealed an upregulation of Rab32 during Schwann cell pyroptosis. Furthermore, the effect of Rab32 on the level of ROS in mitochondria in pyroptosis model has also been studied. Finally, the effects of knocking down the Rab32 gene on PNR were assessed, morphology, sensory and motor functions of sciatic nerves, electrophysiology and immunohistochemical analysis were conducted to assess the therapeutic efficacy.

**Results:**

Silencing Rab32 attenuated PNI-induced Schwann cell pyroptosis and promoted peripheral nerve regeneration. Furthermore, our findings demonstrated that Rab32 induces significant oxidative stress by damaging the mitochondria of Schwann cells in the pyroptosis model in vitro.

**Conclusion:**

Rab32 exacerbated Schwann cell pyroptosis in PNI model, leading to delayed peripheral nerve regeneration. Rab32 can be a potential target for future therapeutic strategy in the treatment of peripheral nerve injuries.

**Supplementary Information:**

The online version contains supplementary material available at 10.1186/s12967-024-04999-x.

## Introduction

Peripheral nerve injury (PNI) is a common condition with a prevalence ranging from 1 to 7% in the general population, with higher prevalence rates observed in individuals aged 50 or above [1, 2]. It is a condition caused by trauma, metabolic disorders, and overuse/compression, that can lead to long-term pain, muscle weakness or paralysis, sensory deficits, and loss of coordination [3, 4]. In recent years, many methods have been developed and advanced to treat peripheral nerve injuries [5], such as autologous nerve grafting technique [6], nerve conduits, among others. However, these methods often yield suboptimal results and each of them has drawbacks [7]. Therefore, it is crucial to conduct further investigation into the underlying mechanisms of PNI, making this a highly relevant area of study.

Schwann cells (SCs) are a prevalent type of glial cell that reside in the myelin sheath. Schwann cells play a vital role in providing trophic support and maintaining the speed of nerve conduction [8]. Recent research has shown that healthy SCs also have a significant impact on regenerating nerve fibers following peripheral nerve injury (PNI). This includes their involvement in the removal of degenerated axons and cellular debris, as well as their ability to promote the growth of regenerating axons and nerves through the production of growth factors [9–13]. Unfortunately, under pathological conditions, dysfunctional Schwann cells could compromise their ability to repair peripheral nerves, leading to poorer patient outcomes [14].

Our previous study demonstrated that Schwann cells undergo pyroptosis in response to PNI [15]. Pyroptosis is a specific form of programmed cell death that that can be triggered by various factors, including damage-associated molecular patterns (DAMPs) [16], DNA damage [17], inflammatory factors [18], and reactive oxygen species (ROS). During this process, a multitude of inflammatory factors, including IL-1β and IL-18, are released into the microenvironment, exerting detrimental effects on neuronal cells and impeding peripheral nerve regeneration. However, the precise of mechanisms underlying Schwann cell pyroptosis in peripheral nerve injury (PNI) remain unclear.

Reactive oxygen species (ROS) are a type of oxidizing agents that contain unpaired electrons [6]. Under normal circumstances, they are generated and cleared by the intracellular redox system in a balanced manner [7]. Abnormal ROS accumulation, triggered by tissue damage and the inflammatory response, leads to the progression of inflammation, injury, and ultimately cell death [19]. Previous study showed that suppression of ROS production by PARKIN/PINK-mediated mitophagy can attenuate PNI-induced Schwann cell death [20]. Recent study demonstrated that abnormal accumulation of ROS has been found to be involved in various triggers of pyroptosis [21]. Further investigation is warranted to understand the mechanism of abnormal ROS production and elucidate the role of ROS in PNI-induced Schwann cells pyroptosis.

Ras-associated binding (Rab) proteins are a family of small GTPase proteins that regulate intracellular membrane transport [22]. The Rab32 protein is a member of the small GTPase protein family, and it plays a crucial role in regulating communication between cellular organelles and transporting substances within cells [23]. Rab32 protein has been found to have a close relationship with mitochondria, such as organelle positioning. Rab32 protein is involved in regulating the transport and positioning of intracellular membranes, including targeting mitochondria to specific regions within the cell [24]. Recent studies have shown that Rab32 is up-regulated in acute neuroinflammation [25]. It also plays a crucial role in controlling the fusion and fission processes of mitochondria [26, 27], which are directly involved in regulating the production of ROS. However, the specific role and underlying mechanism of Rab32 in PNI-induced Schwann cell pyroptosis have yet to be elucidated.

This study aimed to validate the role of Rab32 protein on PNI-induced Schwann cell pyroptosis and investigate the underlying mechanisms, focusing specifically on the interconnections among Rab32, ROS, and pyroptosis.

## Methods and materials

### Materials and reagents

Antibodies were as follows: GSDMD antibody (Lot: #E9S1X) was purchased from Cell Signaling Technology (Massachusetts, USA). Caspase-1 (Lot: #ab179515) and S100 antibodies (Lot: #ab52642) were purchased from Abcam (Cambridge, England, UK). NLRP3 (Lot: # T55651) and Rab32 (Lot: # TD9825) was purchased from Abmart (Shanghai, China).

Reagents were as follows: Rat IL-1β (Lot: #E-EL-R0012c) and IL-18 (Lot: #E-EL-R0567c) ELISA Kits were obtained from Elabscience (Shanghai, China). Lipopolysaccharides (LPS, Lot: #L4391-1MG) and adenosine triphosphate disodium (ATP, Lot: #A2383-5G) was purchased from Sigma-Aldrich. We obtained Mitoquinone mesylate (MitoQ, Lot: # HY-100116A) from MedChemExpress (Monmouth Junction, NJ, USA).

### The construction of the PNI model

Male Sprague–Dawley rats were housed in temperature- and humidity-controlled rooms. The rats were anesthetized before surgery. The left sciatic nerve was isolated and crushed according to previous study [28]. In the Sham group, the left sciatic nerve was exposed without damage, followed by suturing of the muscle layer and skin. Rat sciatic nerves were harvested from a location precisely 1 cm distal to the site of injury in the PNI group, and from a similar location at the sham group. These nerves were then used in subsequent experiments.

Rat nerves were harvested for Toluidine Blue (TB) staining and transmission electron microscopy analysis. The bilateral gastrocnemius muscles of each rat were harvested at the 12th week post-surgery for H&E and Masson staining, as well as weighing.

MitoQ was dissolved in a 1:1 ethanol–water mixture to a concentration of 200 mg/mL and dissolved in 1 mL of 0.9% sterile NaCl and administered intraperitoneally (i.p.) once a day for 4 weeks [29, 30].

### Cell culture

RSC96 cells were purchased from the Cell Bank of the Chinese Academy of Sciences and cultured in Dulbecco’s modified Eagle’s medium (DMEM, Servicebio, Wuhan, China) with 10% fetal bovine serum (FBS, GIBCO) and 1% antibiotics (100 µg/ml penicillin and 100 µg/ml streptomycin). The Schwann cell pyroptosis model was established by treating the cells with LPS (1 μg/mL) for 4 h, followed by a 6-h stimulation with ATP (5 mM). Based on previous reports, Schwann cells were treated with 500 nm MitoQ for 24 h, followed by subsequent experiments [31].

### Immunofluorescence staining

Fresh sciatic nerve segments were fixed in embedding medium and permeabilized using 0.3% Triton X-100 (Servicebio, Wuhan, China). The sections were then blocked and incubated overnight with primary antibodies against NLRP3. Afterwards, the samples were incubated with goat secondary antibodies for 1 h and stained with 4′,6-diamidino-2 phenylindole (DAPI, Lot: #GDP1024, Servicebio, Wuhan, China). Representative images were captured using a fluorescence microscope.

### RNA sequencing

Total RNA of nerve tissue was isolated using TRIzol reagents. These samples were subjected to library construction and sequencing according to manufacturer’s instructions. A q value < 0.05 and foldchange > 2 and < 0.5 were set as the thresholds for significantly differential expression.

### Immunohistochemistry (IHC)

The neural tissues are first fixed with a 4% paraformaldehyde solution and subsequently embedded in paraffin wax. The tissue sections were immunestained using the Rab32 antibody. Images were then obtained with the Leica microscope (Leica microsystems, Germany).

### Mitochondrial membrane potential (ΔΨ)

JC-1 (5,5′,6,6′-tetrachloro-1,1′,3,3′-tetraethylbenzimi-dazolylcarbocyanine iodide) (Lot: #C2003S, Beyotime, Shanghai, China) was used to stain Schwann cells for assessing the cellular mitochondrial membrane potential following the instructions provided by the manufacturer.

### Cellular ROS detection

Schwann cells that underwent treatment were stained with a 10 µM concentration of DCFH-DA, a fluorescent probe for reactive oxygen species (ROS) obtained from Beyotime (China). The staining was performed for 30 min in a dark environment, and the cells were subsequently observed under a fluorescence microscope.

### Mitochondrial ROS detection

Mitochondrial ROS (mtROS) levels in living Schwann cells were measured using the MitoSOX (Invitrogen) assay following the manufacturer’s instructions. Specifically, cells were seeded in a confocal Dish at a density of 5 × 10^5^/ml in each group. After different treatments, the cells were stained with the MitoSOX probe at a final concentration of 5 μM and incubated at 37 °C in the dark for 10 min. Subsequently, the cells were thoroughly washed with PBS, and the mtROS activity was analyzed.

### Lactate dehydrogenase (LDH) assay

According to the instructions, the supernatants of the different treatment groups were collected. Then, 120 μL of the supernatants were transferred to a new 96-well plate and 60 μL of the working solution was added to each well. The plate was incubated for 25 min at room temperature, followed by measurement of absorbance at 490 nm using a microplate reader (Thermo Fisher Scientific, USA). In addition, the maximum LDH release control, consisting of 20 μl of 10 × lysis solution, was added 45 min before the working solution.

### Silencing Rab32 in vitro and in vivo using Adeno-associated virus (AAV)

Short-hairpin small interference RNA (shRNA) were purchased from Genomeditech (Shanghai, China). These target sequences were as follows: Negative control (NC): 5′- TTCTCCGAACGTGTCACGT-3′, shRNA1: 5′-AGACCCGAGAGCACCTCTTTA-3′, shRNA2: 5′-AGCATTTGTAGTCTTTGATAT-3′ and shRNA3: 5′-CTGTCCTTCTAGCTAACAAAT-3′. Rab32 shRNA AAVs were prepared according to the manufacturer's instructions. Rab32 shRNA AAV (AAV. shRab32) have used to infect Schwann cells for 24 h, then Schwann cells were treated with LPS/ATP for pyroptosis. The AAV. shRab32 was injected below the epineurosis using a microscope without damaging the fasciculum in vivo [32].

### Western blotting

The total protein sample was separated via SDS-PAGE and subsequently transferred onto a PVDF membrane. The PVDF membrane was then incubated overnight at 4 °C with primary antibodies, followed by a 1-h incubation with a secondary antibody.

### Real-time PCR

Total RNA was extracted using TRIzol reagent (Lot: # 9108, Takara, Japan), and cDNA was obtained after reverse transcription with a Prime Script RT Reagent Kit (Lot: # 11214ES60, Yeasen, China). The primers sequences were as follows: NLRP3 forward primer: 5′-CTCTGCATGCCGTATCTGGT-3′, NLRP3 reverse primer: 5′- GACGTGCATGCATCATTCCA-3′. Rab32 forward primer: 5′-TGAAACCTCTGCCAAGGATAACA-3′, Rab32 reverse primer: 5′-AGGATCCAGAGGGAGACACC-3′. β-actin forward primers: 5′-CATGTACGTTGCTATCCAGGC-3′, β-actin reverse primer: 5′- CTCCTTAATGTCACGCACGAT- 3′.

### Enzyme-linked immunosorbent assay (ELISA)

The concentration of IL-18 and IL-1β in the culture medium of Schwann cells was measured using an ELISA kit following the manufacturer's instructions.

### Transmission electron microscopy (TEM) analysis

The nerve samples were immersed in an electron microscopy fixative, and the sections were subsequently analyzed using TEM (JEOL, Tokyo, Japan). The number and average diameter of myelinated axons, the thickness of the myelin sheath, and the G ratio (%) were evaluated from TEM micrographs using ImageJ software (NIH, Bethesda, MD, USA).

### Functional assessment of nerve regeneration

The sciatic functional index (SFI) is a widely used method for assessing behavioral and functional recovery following sciatic nerve injury in rodents [33]. In this method, a rat's hind paw is coated with black ink, and the rat is then allowed to traverse a narrow pathway, leaving footprints on a white paper. The formula for calculating SFI is as follows: SFI = (EPL–NPL) × − 38.3/NPL + (ETS–NTS) × 109.5/NTS + (EIT–NIT) × 13.3/NIT–8.8. Here, EPL represents the distance from the heel to the third toe, NPL represents the distance from the heel to the third toe in the control group, ETS represents the distance from the first toe to the fifth toe, NTS represents the distance from the first toe to the fifth toe in the control group, EIT represents the distance from the second toe to the fourth toe, and NIT represents the distance from the second toe to the fourth toe in the control group.

The Von frey test was performed according to the previously described protocol to measure the paw withdrawal threshold [34].

Electrophysiological analysis was conducted to capture electrical signals following established protocols [35]. Briefly, the stimulating electrode was inserted into the proximal end of the sciatic nerve, and a conductive tube was subsequently wrapped around the affected nerves. The electrode was connected to an external stimulator (MD3000-C, Zhenghua, Anhui, China). Pulse stimulation with a frequency of 1 Hz, stimulation intensity of 1 mA, and duration of 0.2 ms was utilized. An additional signal receiving electrode was positioned on the lateral plantar surface of the stimulated leg. The distance between the stimulating electrode and the recording electrode was measured. The peak amplitude of the compound muscle action potential (CMAP) and CMAP onset latency were calculated using previously published methods [36].

### Statistical analyses

The data are represented as mean ± standard deviation (S.D.). Statistical analyses were performed using GraphPad Prism software. One-way ANOVA analysis and Student's t-test were used to evaluate differences between groups. A p-value of less than 0.05 was considered statistically significant.

## Results

### Peripheral nerve injury (PNI) induced pyroptosis and upregulated Rab32 expression

**Fig. 1:**
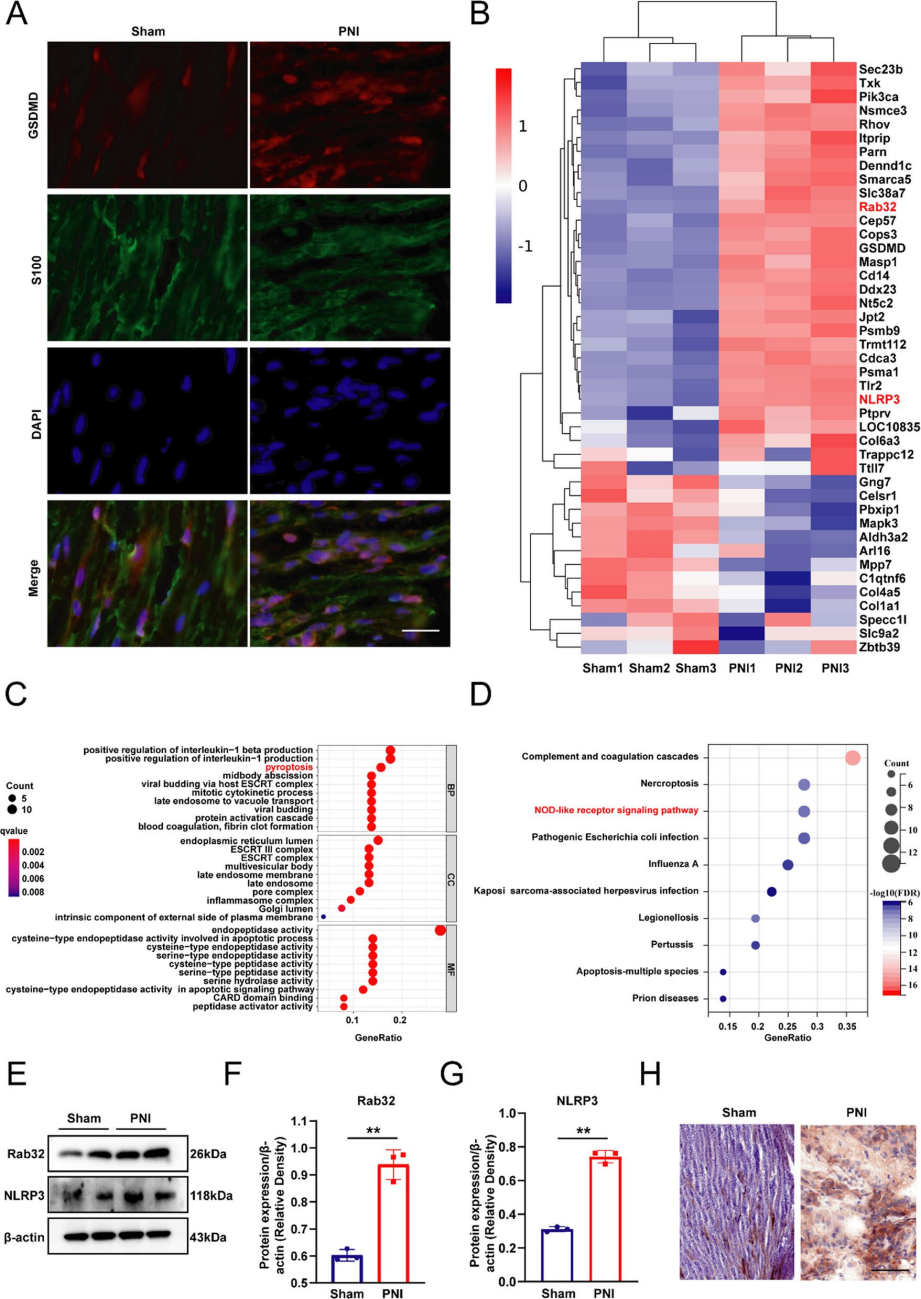
PNI induced pyroptosis and upregulated Rab32 expression. **A** Immunofluorescence staining for S100 (green) and GSDMD (red) in nerve tissues. Nuclei were labeled using DAPI (blue). Scale bar = 20 µm. **B** Heat map summarizing the DEGs related to Rab32 and pyroptosis-related gene. **C** The GO biological process enrichment analysis of DEGs. **D** The KEGG biological process enrichment analysis of DEGs. **E**–**G** Protein expression levels and quantitative results of Rab32 in Sham and PNI groups. (H) Immunohistochemical staining of Rab32 in peripheral nerve tissue. **P < 0.01. Scale bar = 50 µm

As shown in Fig. 1A, compared to the sham group, the level of the pyroptosis-related protein GSDMD was significantly increased in the injured neural tissue. In addition, the expression of the Rab32 and pyroptosis-related gene NLRP3 is significantly increased in the PNI group from the Heat Map (Fig. 1B). We conducted an analysis on the differentially expressed genes (DEGs) in two groups of data and found that pyroptosis and NOD-like receptor signaling pathway were enriched in the GO analysis (Fig. 1C) and KEGG analysis (Fig. 1D), respectively. Protein expression levels and quantitative results of Rab32 in Sham and PNI groups were shown in Fig. 1E–G, the expression level of Rab32 in the PNI group significantly exceeded that of the Sham group, confirming the elevated expression of Rab32 protein following PNI. The immunohistochemical results also confirmed that the expression of Rab32 protein is significantly upregulated after peripheral nerve injury (Fig. 1H).

### Rab32 regulated mitochondrial function and ROS production

**Fig. 2:**
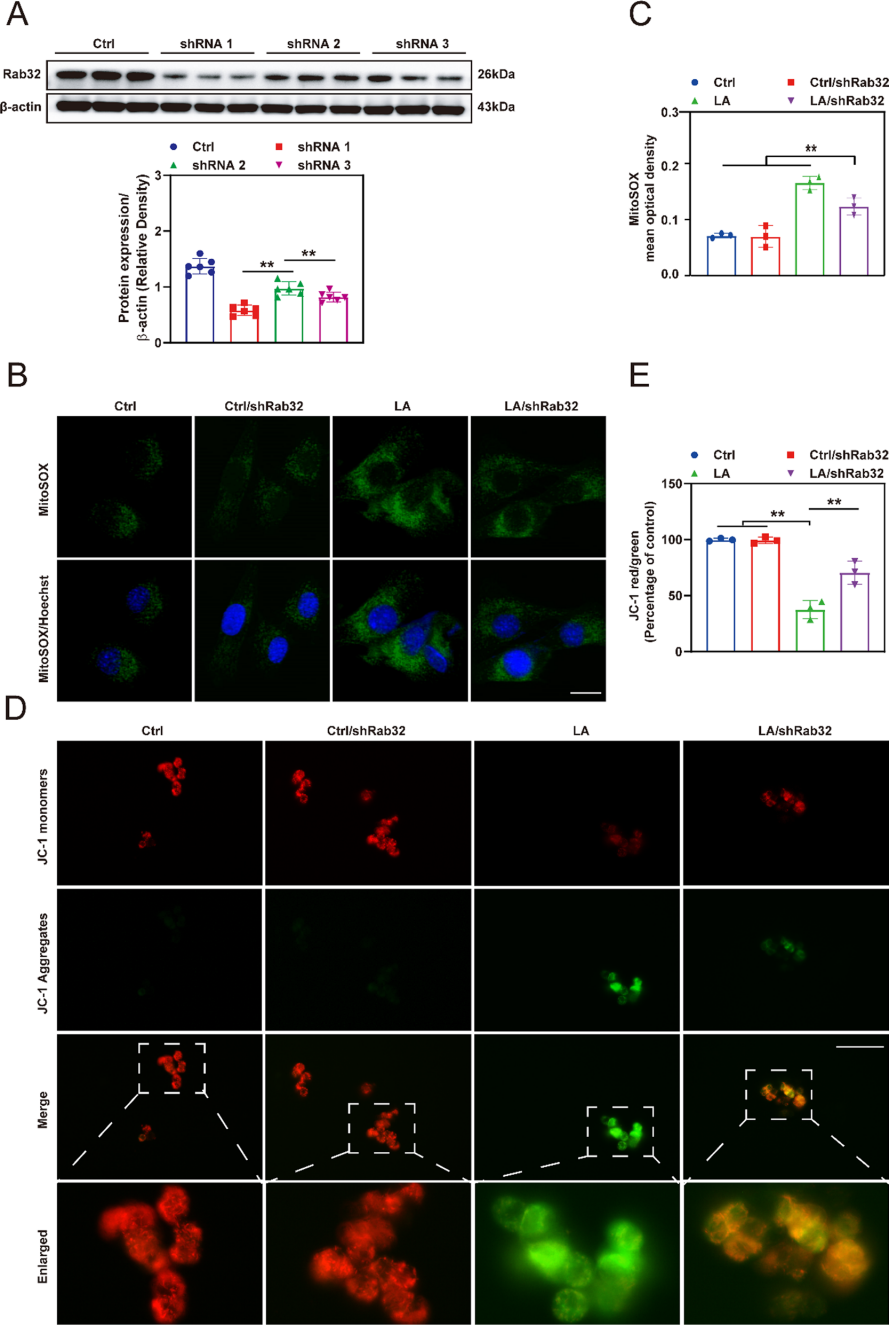
Knocking down of Rab32 significantly reduces the mitochondrial ROS content in Schwann cells in vitro. **A** Western blotting accessed the expression of Rab32 after Schwann cells were infected with AAV. shRab32 for 24 h. Schwann cells were infected with AAV. shRab32 for a duration of 24 h, followed by induction of cellular pyroptosis using LPS and ATP. **p < 0.01 compared to the shRNA 2 group. *p < 0.05 compared to the shRNA 2 group. **B** and **C** Representative immunofluorescence images and quantitative analysis of the level of mitochondrial ROS using MitoSOX staining. Scale bar = 20 µm. **p < 0.01 compared to the LA/shRab32 group. *p < 0.05 compared to the LA/shRab32 group. **D** The representative images of mitochondrial membrane potential using JC-1 staining. Scale bar = 50 µm. **E** Quantification of the red-to-green (Percentage of control). Red: JC-1 monomers, green: JC-1 aggregates. **p < 0.01 compared to the LA group. *p < 0.05 compared to the LA group

We initially validated the knockdown efficiency of three Rab32 sequences, and subsequently selected the sequence with the lowest knockdown efficiency for further experimentation (Fig. 2A). Subsequently, MitoSOX (Fig. 2B, C) and JC-1 (Fig. 2D, E) were conducted to assess the condition of the mitochondria in each group. The results indicated that the mitochondria ROS levels in the LA (LPS/ATP)/shRab32 group were significantly lower than those in the LA group, but still substantially higher than those in the Ctrl group and Ctrl/shRab32 group. Furthermore, as shown in Additional file 1: Fig. S1A, the mitochondria in the LA group showed a wrinkled shape, accompanied by blurred and significantly reduced cristae. Compared to the LA group, the cristae of mitochondria in the LA/shRab32 group were clearer. Further quantitative results showed that the LA/shRab32 group exhibited significantly improved mitochondrial number (Additional file 1: Fig. S1B) and average volume (Additional file 1: Fig. S1C) compared to the LA group, indicating a protective effect of Rab32 knockdown on mitochondria. Pyroptosis results in the release of lactate dehydrogenase (LDH) from cells into the extracellular space, and the extent of pyroptosis can be indirectly assessed by measuring the LDH concentration in the cell culture medium [37]. Consistent with previous results, knocking down Rab32 levels can reduce LDH levels in LPS/ATP-treated Schwann cells, further demonstrating that Rab32 can exacerbate pyroptosis (Additional file 1: Fig. S2). We then used transmission electron microscopy to observe the morphology of normal and pyroptotic Schwann cells (Additional file 1: Fig. S3). We then used transmission electron microscopy to observe the morphology of normal and pyroptotic Schwann cells. It was observed that pyroptotic Schwann cells had ruptured cell membranes, which is also one of the characteristic features of pyroptosis [38].

### ROS scavengers MitoQ inhibit pyroptosis in Schwann cells

**Fig. 3:**
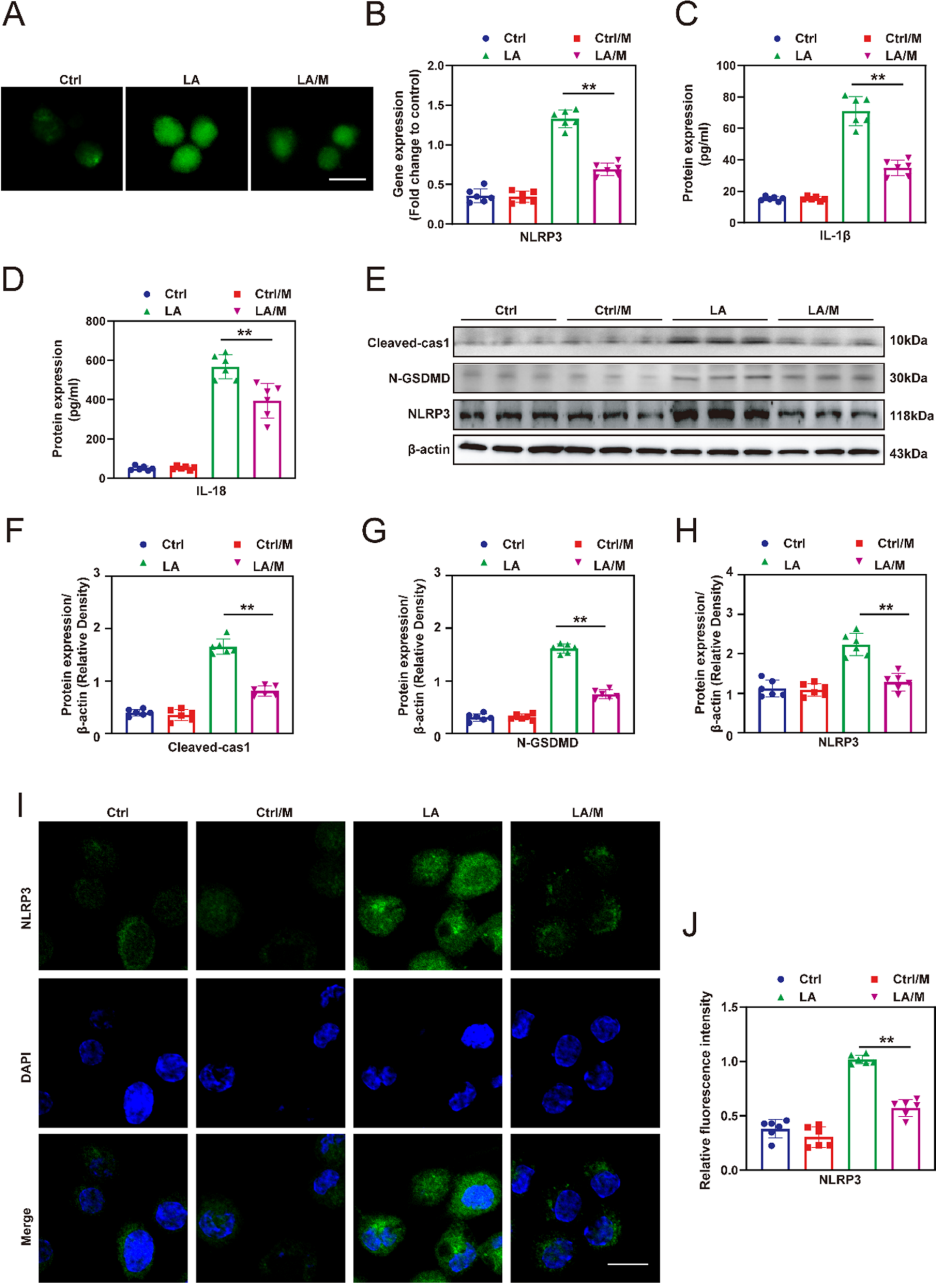
The impact of MitoQ on pyroptosis in vitro. **A** The level of ROS was assessed by DCFH-DA staining. **B** Expression of NLRP3 mRNA was analyzed by real-time PCR. **C** and **D** The levels of IL-1β and IL-18 in the culture medium were determined by ELISA. **E** The expression of Cleaved-cas1, N-GSDMD and NLRP3 protein was evaluated by western blotting, and representative bands are shown. **F**–**H** The histograms show the expression of the Cleaved-cas1, N-GSDMD and NLRP3 proteins in according to the semi-quantitative analysis of the bands. **I** Representative immunofluorescence images showing the location and level of NLRP3 in Schwann cells. **J** The quantitative analysis of mean fluorescence intensity (MFI) of NLRP3 in each group. Green: NLRP3, blue: DAPI. Scale bar = 20 µm. **p < 0.01, *p < 0.05

The mitochondrial ROS scavenger, MitoQ, was used to examine the impact of mitochondrial ROS levels on the level of pyroptosis in Schwann cells. The DCFH-DA tests (Fig. 3A) shows that MitoQ (M) effectively reduces the ROS level in Schwann cells treated with LPS/ATP (LA). The real-time PCR results showed that the gene expression of NLRP3 (Fig. 3B) in the LA group was significantly downregulated compared with the LA group, although they were slightly higher than those in the Ctrl and Ctrl/M groups. Furthermore, the enzyme-linked immunosorbent assay (ELISA) was employed to assess the levels of IL-1β (Fig. 3C) and IL-18 (Fig. 3D) in each group, which allowed for the evaluation of the pyroptosis level of Schwann cells in each group, the results showed that the IL18 and IL1β levels in the LA/M group were significantly lower than those in the LA group (p < 0.05). Subsequently, western blotting was used to assess the levels of pyroptosis related proteins Cleaved-cas1, N-GSDMD and NLRP3 in each group. The results showed that the levels of these proteins in the LA/M group were significantly lower than those in the LA group, but still higher than those in the Ctrl group and Ctrl/M group (Fig. 3E–H) (*p* < 0.05). Finally, immunofluorescence staining was performed to determine the localization of NLRP3, a specific marker for pyroptosis (Fig. 3I). The mean fluorescence intensity (MFI) of NLRP3 was significantly lower in the LA/M group group compared to the LA group (Fig. 3J) (*p* < 0.05). These results provide evidence that the application of MitoQ significantly inhibits pyroptosis in Schwann cells in vitro.

### Rab32 involved in PNI-induced pyroptosis in vivo.

**Fig. 4:**
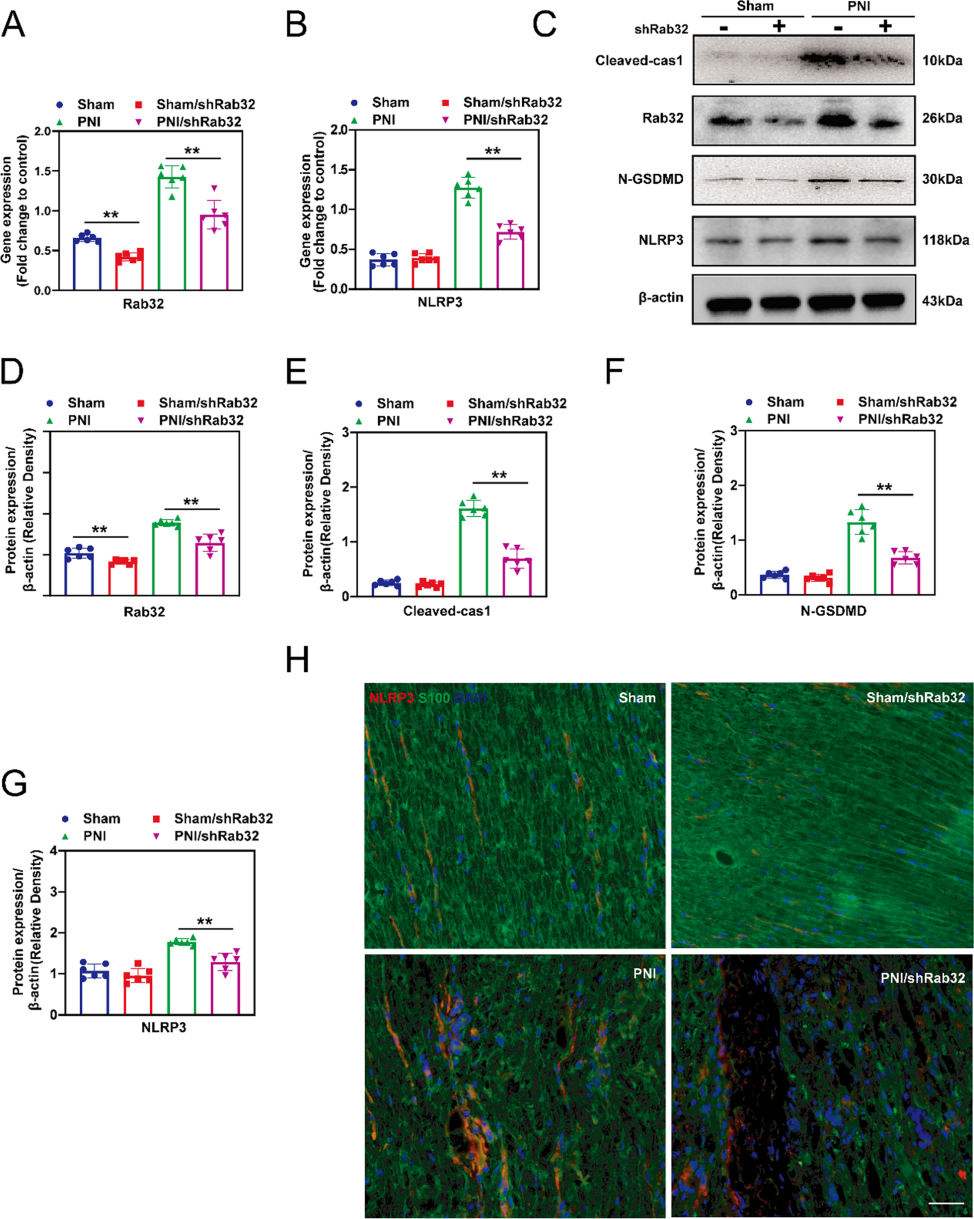
Knockdown of Rab32 attenuates PNI-induced pyroptosis in vivo. Peripheral nerves tissue was infected with AAV.shRab32, followed by PNI treatment. These samples were harvested on the 3rd day postoperatively. **A** and **B** Expression of NLRP3 and Rab32 mRNA was analyzed by real-time PCR. **C** The expression of Cleaved-cas1, Rab32, N-GSDMD and NLRP3 protein was evaluated by western blotting, and representative bands are shown. **D**–**G** The histograms show the expression of the Rab32, N-GSDMD, Cleaved-cas1 and NLRP3 proteins in according to the semi-quantitative analysis of the bands. **H** Immunofluorescent analysis revealed the localization of NLPR3 in Schwann cell within the tissue of the peripheral nervous. Red: NLRP3, Green: S100 (Schwann cell marker), and bule: DAPI. Scale bar = 100 μM. **p < 0.01, *p < 0.05

After pyroptosis, a large amount of inflammatory substances are released, which hinders the regeneration of surrounding nerves. Therefore, reducing the level of pyroptosis in the tissue is of great significance for the regeneration of peripheral nerves, especially Schwann cells pyroptosis. We first verified the effectiveness of AAV in vivo to knock down levels of Rab32. The results showed that the Rab32 gene (Fig. 4A) and protein levels (Fig. 4D) in the PNI/shRab32 group were significantly lower than in the PNI group, indicating successful knockdown of Rab32 in the PNI/ shRab32 group. The real-time PCR results showed that the gene expression of NLRP3 in the PNI/shRab32 group was significantly downregulated compared with the PNI group, although it was slightly higher than those in the Sham and Sham/shRab32 groups (Fig. 4B). The subsequent western blotting results (Fig. 4C) show that the levels of pyroptosis-related protein, Cleaved-cas1, N-GSDMD and NLRP3 were significantly reduced in the PNI/ShRab32 group compared with those in the PNI group, which were still higher than those in the Sham and Sham/shRab32 groups, indicating that the reduction in Rab32 protein levels alleviates the level of pyroptosis in vivo (Fig. 4E–G). In addition, immunofluorescence staining was used to detect the localization of the specific pyroptosis marker, NLRP3, and specific Schwann cell marker, S100. The MFI of NLRP3 in the PNI/shRab32 group was significantly lower than that in the PNI group, and this is consistent with the previous results (Fig. 4H). The morphology of mitochondria in each group was also evaluated (Additional file 1: Fig. S4A), and the results showed that the mitochondrial morphology in the PNI/shRab32 group was superior to that in the PNI group. This was manifested by severe mitochondrial shrinkage and a significant reduction in mitochondrial cristae in the PNI group, while the mitochondrial morphology in the PNI/shRab32 group was still inferior to that in the Sham group and the Sham/shRab32 group. Further quantitative results showed that the PNI/shRab32 group exhibited significantly improved mitochondrial number (Additional file 1: Fig. S4B) and average volume (Additional file 1: Fig. S4C) compared to the PNI group, indicating a protective effect of Rab32 knockdown on mitochondria.

Meanwhile, we observed the effect of MitoQ on PNI-induced pyroptosis in vivo. The results showed that the in vivo application of MitoQ reduced the level of pyroptosis induced by PNI (Additional file 1: Fig. S5).

### Rab32 negatively regulates peripheral nerve function in the post-operative period

To investigate the impact of Rab32 protein on peripheral nerve regeneration, we first used adeno-associated virus to knock down the levels of Rab32 in vivo. Then, we observed its effect on the regeneration of the rat sciatic nerve injury.

**Fig. 5:**
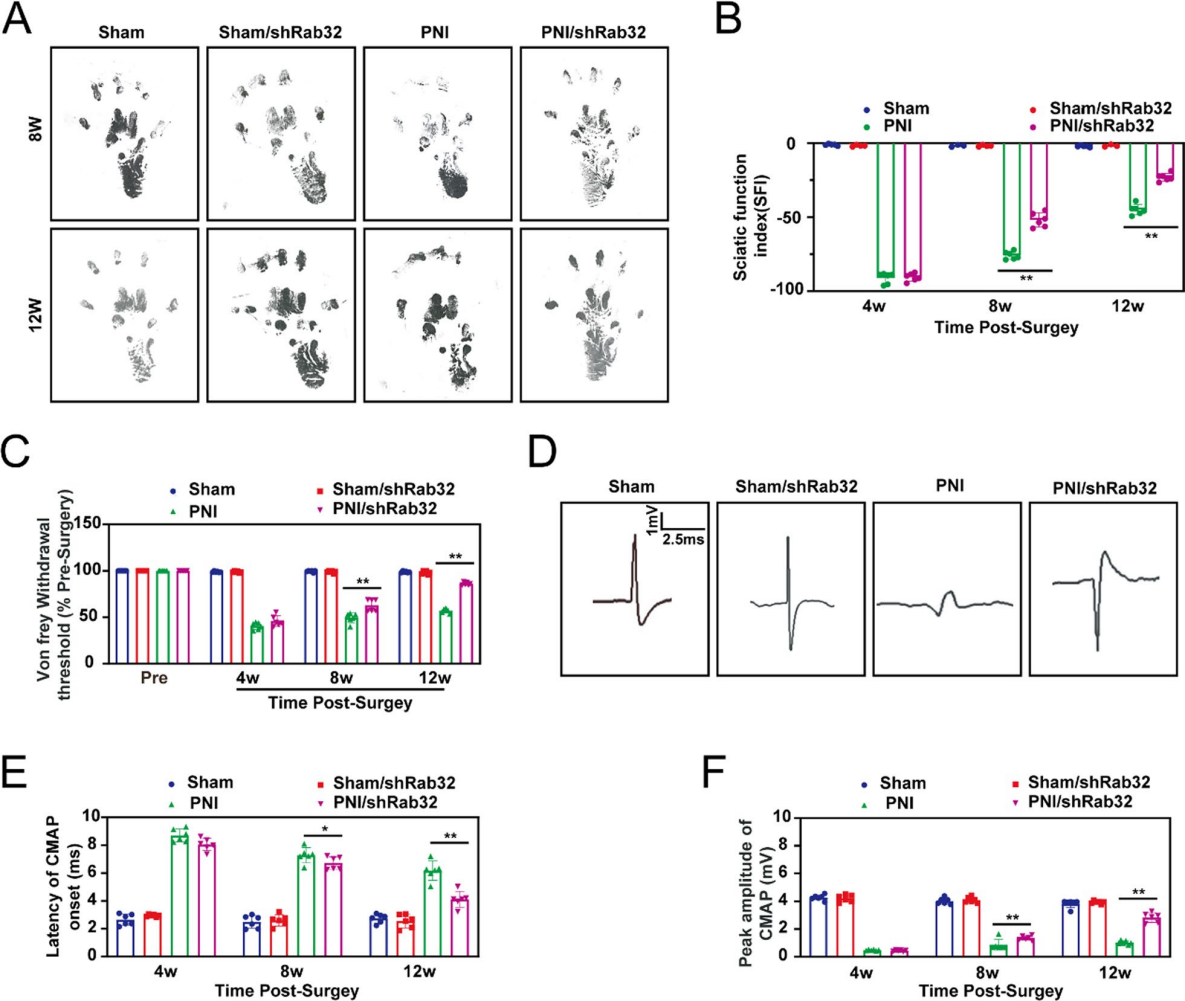
Silencing Rab32 improved the function of peripheral nerves in the postoperative period. The function of peripheral nerves had been evaluated during pre-surgery, and the 4th, 8th, and 12th postoperative weeks. **A** and **B** The representative image of footprints and quality analysis in Sciatic Functional Index (SFI) assay. **C** The relative level of Von Frey withdrawal threshold. **D** The representative image of electrophysiological analysis. **E** and **F** The histograms illustrated the levels of Latency of CMAP onset and peak amplitude of CMAP. **p < 0.01, *p < 0.05

In order to evaluate the potential effects of Rab32 on the recovery of motor function in rats with sciatic nerve injury, we assessed the recovery of motor function by measuring SFI at 4, 8, and 12 weeks post-surgery (Fig. 5A, B). The results showed that there was no significant difference between the PNI group and the PNI/shRab32 group at the 4th week post-surgery, which is consistent with previous findings. This lack of difference might be due to the short duration of nerve repair initiation at this stage. However, at the 8th and 12th week, the SFI results in the PNI/shRab32 group were significantly better than those in the PNI group, indicating that downregulation of Rab32 levels can promote the recovery of sciatic nerve motor function.

The mechanical sensitivity test (Von Frey test) is a test that applies thin calibrated plastic filaments to the plantar surface of the hind paws. Different thicknesses or stiffnesses of Von Frey filaments are used to determine the threshold for eliciting withdrawal responses. In the study, the Von Frey test is used to evaluate the functional recovery after nerve crush at 4, 8, and 12 weeks post-surgery (Fig. 5C). No significant differences were observed at week 4, but the Von Frey test showed that the rats in the PNI/shRab32 group had faster functional recovery at 8 and 12 weeks post-surgery compared to the PNI group.

Electrophysiological tests are key indicators for evaluating recovery after sciatic nerve injury. In our study, electrophysiological indicators were evaluated at 4, 8, and 12 weeks post-surgery (Fig. 5D). The results showed that at the 8th and 12th weeks post-surgery, the CMAP latency and nerve conduction velocity indicators of the PNI/shRab32 group were significantly better than those of the PNI group (Fig. 5E, F), confirming that knocking down Rab32 levels can promote peripheral nerve regeneration.

### Rab32 delayed promotes the regeneration of peripheral nerves in vivo

**Fig. 6:**
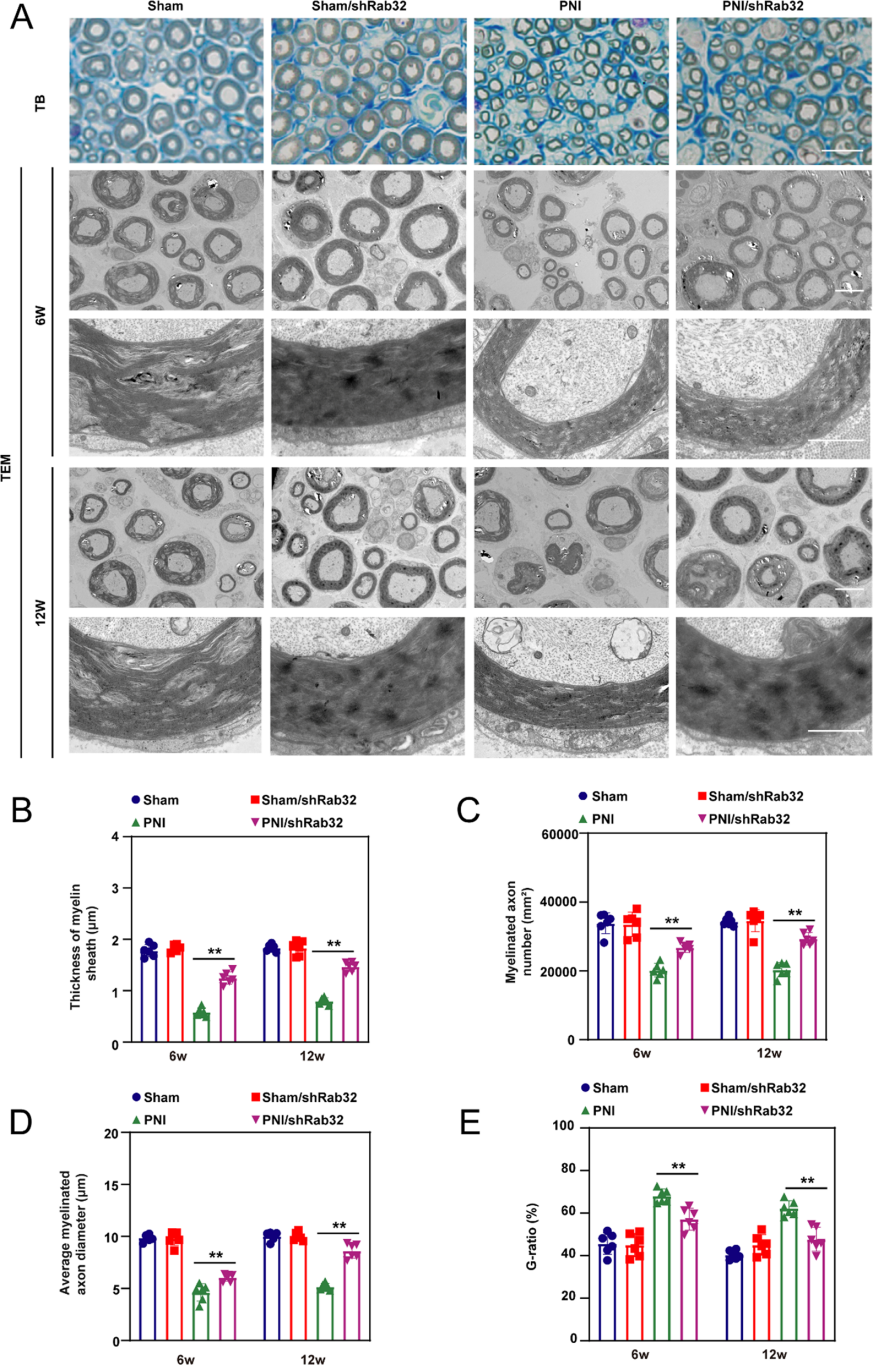
Rab32 downregulation improve promotes the regeneration of peripheral nerves in vivo. **A** TB staining examined the thickness of the myelin sheath in peripheral nerves at 12th postoperative week, Scale bar = 10 µm. TEM analysis evaluated regeneration of peripheral nerves at 6th and 12th postoperative week. Scale bar = 5 µm (upper), and 1 µm (lower). **B** Thickness of myelin sheath. **C** Number of myelinated axons. **D** Average myelinated axons diameter. **E** G-ratio. **p < 0.01, *p < 0.05

Toluidine blue (TB) staining and TEM were used to evaluate remyelination of the regenerated nerves (Fig. 6A). According to the TB staining results, the myelin sheath thickness in the PNI/shRab32 group is significantly better than that in the PNI group, but worse than that in the Sham group and Sham/shRab32 group. The neuro-electron microscopy results at 6 and 12 weeks were evaluated. The findings indicate that the thickness of myelin sheath, number of myelinated axons, and myelinated axon diameter in the PNI/shRab32 group were superior to those in the PNI group (Fig. 6B–D), while they were still lower than those in the Sham group and Sham/shRab32 group (*p* < 0.05).

The G-ratio serves as an indicator of the ratio between neural axons and myelin sheath. A lower G-ratio value implies a greater abundance of myelin sheath, which facilitates efficient nerve regeneration. In this study, the results demonstrate significantly lower G-ratio values in the PNI/shRab32 group compared to the PNI group (*p* < 0.05) (Fig. 6E). Consequently, it can be inferred that decreased Rab32 protein levels contribute to increased myelin sheath formation, ultimately facilitating the regeneration of peripheral nerves.

### Rab32 aggravated PNI induced denervation drives gastrocnemius muscle atrophy

**Fig. 7:**
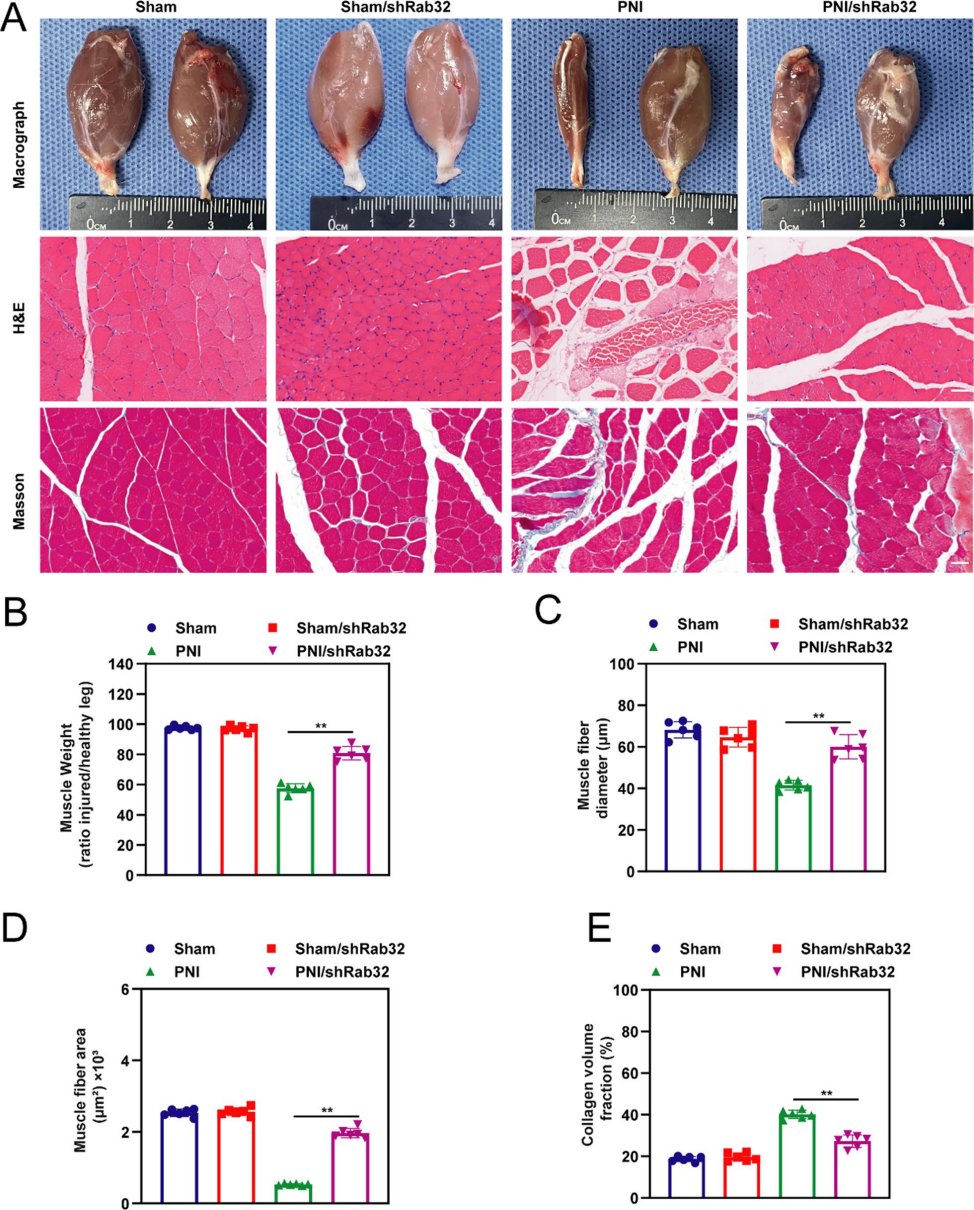
Silencing Rab32 improve PNI induced Denervation drives Gastrocnemius muscle atrophy. The severity of denervation-induced gastrocnemius muscle atrophy was evaluated at 12 weeks postoperatively. **A** The gastrocnemius muscle was assessed for pathological changes through macroscopic analysis (upper) as well as H&E (middle) and Masson staining (lower). **B**–**E** The degree of recovery in atrophied gastrocnemius muscles were accessed by muscle weight (ratio injured/healthy limb), muscle fiber area, muscle fiber diameter and the collagen volume fraction (%), respectively. **p < 0.01, *p < 0.05. Scale bar = 40 µm

Target muscle assessment is one of the key factors in evaluating the regeneration of the sciatic nerve. When the target muscle loses neural innervation, there may be varying degrees of muscle fiber atrophy, along with a potential increase in collagen volume. Target muscle assessment typically involves analyzing muscle morphology and histology. In the study, at 12 weeks post-operation, samples were taken from both sides of the gastrocnemius muscles of each rat, followed by the evaluation of their gross morphology and histological results (Fig. 7A). Results showed that the PNI/shRab32 group exhibited a significantly fuller external appearance of the gastrocnemius muscle compared to the PNI group. This finding is consistent with the subsequent results regarding the wet weight ratio between the affected and unaffected sides of the gastrocnemius muscle (Fig. 7B). Nonetheless, the PNI/shRab32 group's wet weight ratio remains lower than those of the Sham group and Sham/shRab32 group. The results of HE and Masson staining showed that the muscle fibers in the PNI/shRab32 group of rats were more abundant compared to the PNI group. The muscle fiber diameter (Fig. 7C) and fiber area (Fig. 7D) in the PNI/shRab32 group were significantly higher compared to the PNI group at 12 weeks after surgery, while no significant difference was observed between the Sham group and Sham/shRab32 group. Additionally, the proportion of collagen fibers was significantly lower in the PNI/shRab32 group than in the PNI group (Fig. 7E) (*p* < 0.05).

## Discussion

**Fig. 8:**
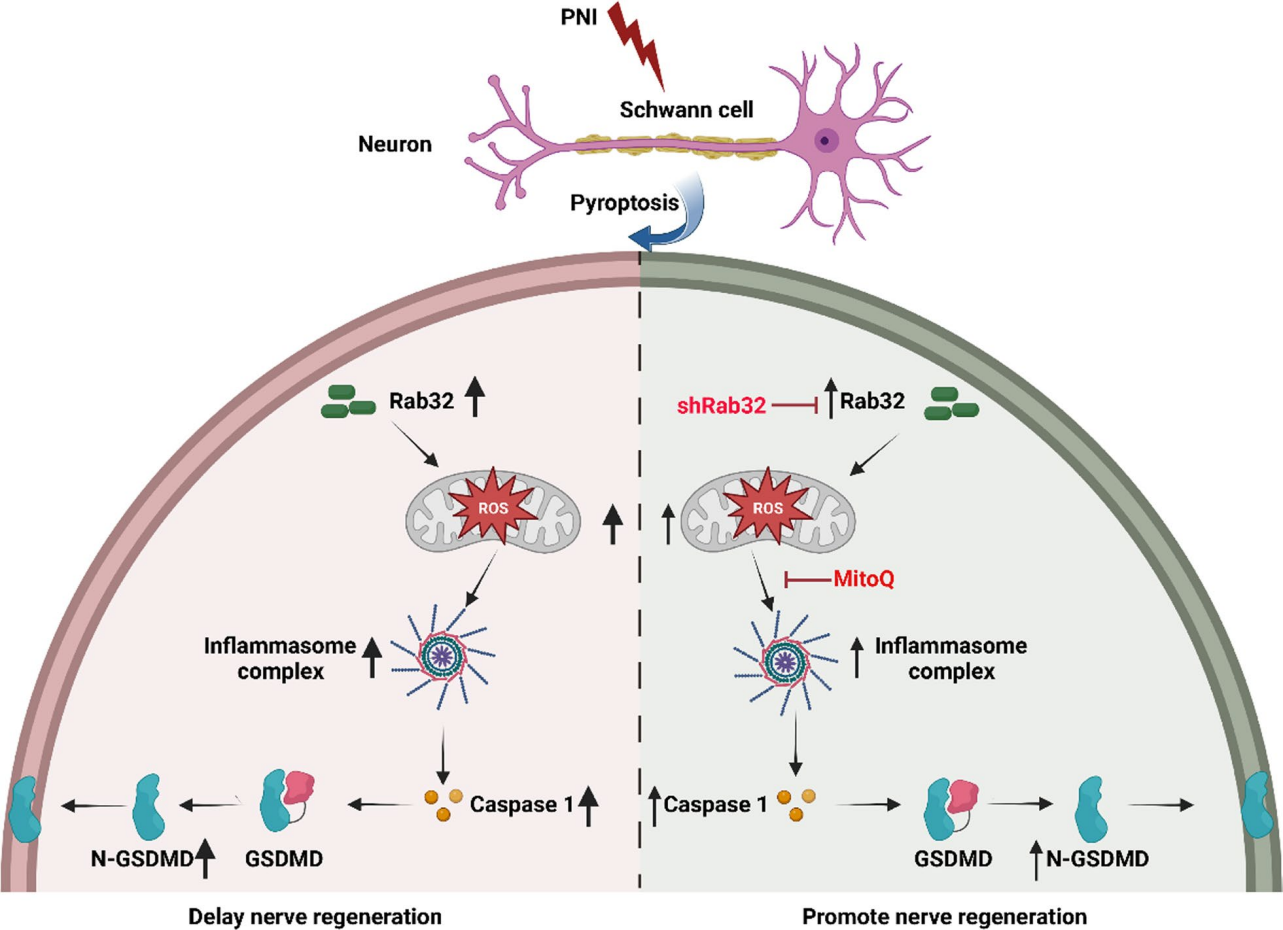
Rab32 facilitates Schwann cell pyroptosis in rats following peripheral nerve injury by elevating ROS levels

In this study, our research confirms that Rab32 plays a pivotal role in peripheral nerve injury (PNI), exacerbating PNI-induced Schwann cell pyroptosis and enhancing inflammatory response. This ultimately leads to the delayed regeneration of peripheral nerves and impaired functionality of the gastrocnemius muscle. Furthermore, in vitro experiments confirmed that downregulation of Rab32 leads to decreased mitochondrial ROS levels and subsequently reduced pyroptosis. The identification of this mechanism opens new perspectives and directions for future research in the field of PNI (Fig. 8).

Schwann cells have great plasticity, which undergo a repair phenotype to promote peripheral nerve regeneration and functional recovery when they receive injury-induced signaling, including c-Jun and Hippo signaling [12, 39–41]. There is increasing evidence that cytokines (such as TNF-α, IFN-γ) [42], mutations in myelin protein zero (MPZ) [43], Acanthamoeba [44] and long-term hyperglycemia conditions [45, 46] induce Schwann cell death, then disrupt peripheral nerve support and neuropathy, and delay peripheral nerve regeneration [5, 47]. Recent study shows that pyroptosis is a major mode of Schwann cell death in diabetic peripheral neuropathy (DPN) [45]. Our previous study and this one showed that Schwann cell pyroptosis impairs peripheral nerve regeneration after peripheral nerve injury, and suppressing Schwann cell pyroptosis could be a novel strategy to promote peripheral nerve regeneration and restore function [15].

Rab GTPase plays an important role in regulating the function of Schwann cells. Previous research demonstrated that the silencing of Rab8a or Rab11a leads to Schwann cell apoptosis and inhibits axonal outgrowth in co-cultured dorsal root ganglion neurons [48]. Su and colleagues showed that Rab27a levels increase during Schwann cell myelination, and that knocking down Rab27a suppressed myelin protein expression and impaired the formation of myelin-like membranes in DRG neurons [49]. Herein, we found that the level of Rab32 was increased during PNI-induced Schwann cell pyroptosis, and suppression of Rab32 could attenuate Schwann cell pyroptosis and promote regeneration of peripheral nerves as well as improve neuron drives gastrocnemius muscle atrophy. These data demonstrated that Rab32 plays a crucial role in peripheral nerve injury, particularly in nerve repair mediated by Schwann cell. It serves as a novel therapeutic target for peripheral nerve injury and repair.

Rab32 protein plays a significant role in regulating mitochondrial dynamics. Previous studies have demonstrated that Rab32 facilitates the migration and invasion of glioblastoma cells by modulating ERK/Drp1-mediated mitochondrial fission [50]. Additionally, Rab32 has been implicated in the regulation of cellular apoptosis and the properties of mitochondria-associated membranes (MAMs) [51]. Furthermore, we discovered that Rab32 regulates Schwann cell pyroptosis through its involvement in ROS production and inflammasome activation. A previous study demonstrated that elevated mitochondrial ROS levels modulate inflammasome signaling pathways, leading to GSDMD cleavage and subsequent induction of pyroptosis in macrophages [52]. Hence, these findings suggest that Rab32 can induce the loss of mitochondrial membrane potential (∆Ψm), enhance mitochondrial ROS production and inflammasome formation, ultimately resulting in Schwann cell pyroptosis. Nevertheless, the specific mechanism underlying Rab32 upregulation in PNI-induced Schwann cell pyroptosis, as well as the role of Rab32 in regulating the mitochondrial membrane potential, requires additional investigation.

These are some limitations. First, the role of Rab32 in Schwann cells induced by PNI should be further confirmed by utilizing Rab32-deficient rat. Second, the novel mechanism responsible for Rab32’s regulation of mitochondrial dysfunction and ROS production in Schwann cell pyroptosis should be further investigated.

## Conclusions

Taken together, Rab32 exacerbated PNI-induced Schwann cell pyroptosis through loss of ∆Ψm and increased ROS production, leading to delayed peripheral nerve regeneration. Therefore, Rab32 silencing appears to be a viable therapeutic strategy to combat PNI and promote peripheral nerve regeneration.

### Supplementary Information

**Additional file 1:** **Figure S1.** The protective effect of Rab32 knockdown on mitochondria. **A** Representative transmission electron microscope image showing mitochondria morphological changes accompanying Schwann cell pyroptosis induced by LPS/ATP. Scale bar = 2 µm. **B** Quantitative data of the average number of mitochondria per unit area. **C** Quantitative data of the average volume of mitochondria per unit area. **p < 0.01 compared to the LA/shRab32 group. *p < 0.05 compared to the LA/shRab32 group. **Figure S2.** Quantification of LDH release indicating Schwann cell damage. **p < 0.01 compared to the LA group. *p < 0.05 compared to the LA group. **Figure S3.** Representative transmission electron microscope image showing morphological changes accompanying Schwann cell pyroptosis induced by LPS/ATP. The black arrows represent the pores on the cell membrane, which is also one of the characteristic signs of pyroptosis. Scale bar = 2 µm. **Figure S4.** The impact of Rab32 on mitochondrial morphology in Schwann cells following peripheral nerve injury. **A** Representative transmission electron microscope image showing mitochondrial morphological changes accompanying Schwann cell pyroptosis induced by PNI. Scale bar = 2 µm. **B** Quantitative data of the average number of mitochondria per unit area. **C** Quantitative data of the average volume of mitochondria per unit area. **p < 0.01 compared to the PNI/shRab32 group. *p < 0.05 compared to the PNI/shRab32 group. **Figure S5.** Evaluation of the effect of MitoQ on pyroptosis in peripheral nerve injury. **A** Western blotting analysis was conducted to evaluate the levels of pyroptosis-associated proteins in nerve tissues. β-actin was used as an internal control. **B**–**D** Levels of pyroptosis-associated proteins were quantified based on semi-quantitative band analysis. **E** Representative immunofluorescence images displaying NLRP3 (red) and S100β (green) in nerve tissues. **p < 0.01 compared to the PNI/M group. *p < 0.05 compared to the PNI/M group. Scale bar = 20 µm.

## Data Availability

All data generated in this study are available within the article.
