# Supplementary material for: Rab32 facilitates Schwann cell pyroptosis in rats following peripheral nerve injury by elevating ROS levels
Source: J Transl Med. 2024 Feb 22;22:194. doi: 10.1186/s12967-024-04999-x (PMC10885539; doi:10.1186/s12967-024-04999-x)
Supplement: Supplementary file 1 — Additional file 1: Figure S1. The protective effect of Rab32 knockdown on mitochondria. A Representative transmission electron microscope image showing mitochondria morphological changes accompanying Schwann cell pyroptosis induced by LPS/ATP. Scale bar = 2 µm. B Quantitative data of the average number of mitochondria per unit area. C Quantitative data of the average volume of mitochondria per unit area. **p < 0.01 compared to the LA/shRab32 group. *p < 0.05 compared to the LA/shRab32 group. Figure S2. Quantification of LDH release indicating Schwann cell damage. **p < 0.01 compared to the LA group. *p < 0.05 compared to the LA group. Figure S3. Representative transmission electron microscope image showing morphological changes accompanying Schwann cell pyroptosis induced by LPS/ATP. The black arrows represent the pores on the cell membrane, which is also one of the characteristic signs of pyroptosis. Scale bar = 2 µm. Figure S4. The impact of Rab32 on mitochondrial morphology in Schwann cells following peripheral nerve injury. A Representative transmission electron microscope image showing mitochondrial morphological changes accompanying Schwann cell pyroptosis induced by PNI. Scale bar = 2 µm. B Quantitative data of the average number of mitochondria per unit area. C Quantitative data of the average volume of mitochondria per unit area. **p < 0.01 compared to the PNI/shRab32 group. *p < 0.05 compared to the PNI/shRab32 group. Figure S5. Evaluation of the effect of MitoQ on pyroptosis in peripheral nerve injury. A Western blotting analysis was conducted to evaluate the levels of pyroptosis-associated proteins in nerve tissues. β-actin was used as an internal control. B–D Levels of pyroptosis-associated proteins were quantified based on semi-quantitative band analysis. E Representative immunofluorescence images displaying NLRP3 (red) and S100β (green) in nerve tissues. **p < 0.01 compared to the PNI/M group. *p < 0.05 compared to the PNI/M group. Scale bar = 20 µm. [file 12967_2024_4999_MOESM1_ESM.docx]

**Rab32 facilitates Schwann cell pyroptosis in rats following peripheral nerve injury by elevating ROS levels**

Jiayi Wang^†1^, Pin Chen^†3^, Guanjie Han^†1^, Yongjie Zhou^†4^, Xingdong Xiang^5^, Mengxuan Bian^1^, Lei Huang^1^, Xiang Wang*^6,7^, Binfeng He*^8,9^, Shunyi Lu*^1,2^

^1^ Department of Orthopedic Surgery, Zhongshan Hospital, Fudan University, Shanghai, China.

^2^ Department of Orthopedic Surgery, The First Affiliated Hospital of Soochow University, Suzhou, Jiangsu, China.

^3^ Department of Neurosurgery, Zhongshan Hospital, Fudan University, Shanghai, China.

^4^ Department of Interventional Radiology, Zhongshan Hospital, Fudan University, Shanghai, China.

^5^ Department of Rehabilitation, Zhongshan Hospital, Fudan University, Shanghai, China.

^6^ Department of Cardiology, The First Affiliated Hospital of Nanchang University, Nanchang, Jiangxi Province, China.

^7^ Department of Cardiology, Zhongshan Hospital, Fudan University, Shanghai, China.

^8^ Department of Pulmonary and Critical Care Medicine, Zhongshan Hospital, Fudan University, Shanghai, China.

^9^ Department of Genel Practice, Xinqiao Hospital, Third Military Medical University, Chongqing, China

^†^ Contributed equally.

Correspondence should be addressed to Binfeng He: ldhbf@126.com


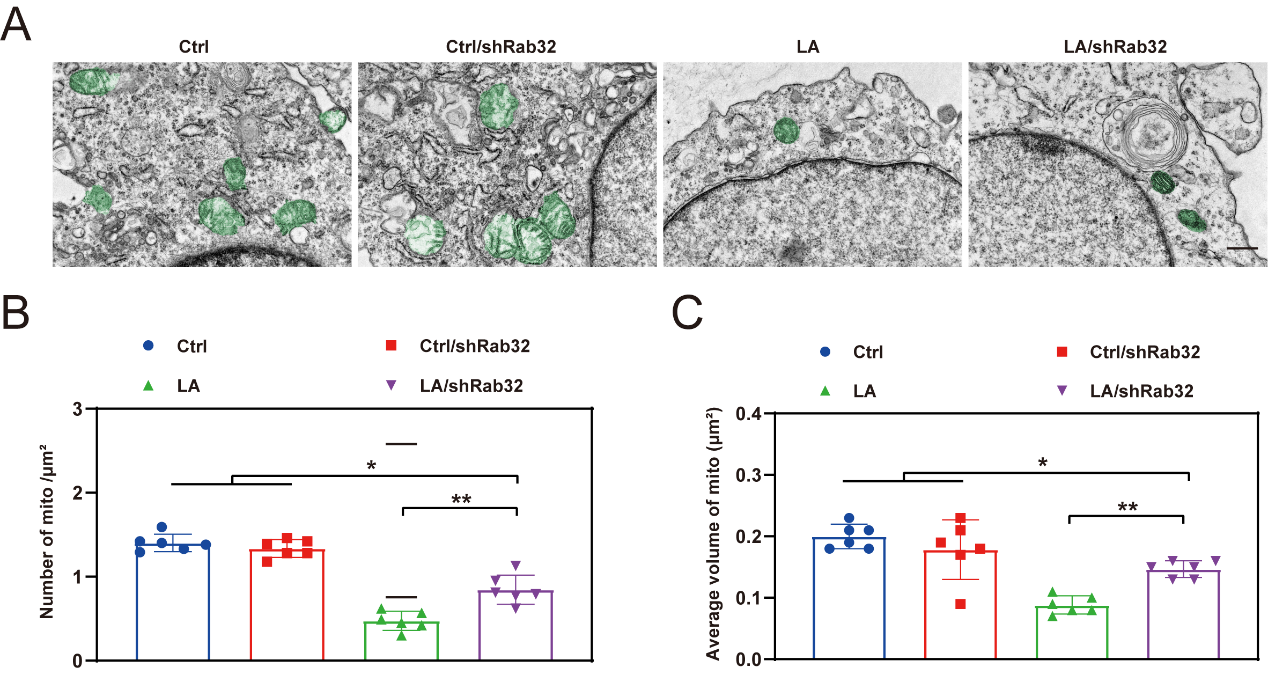


**Figure S1. The protective effect of Rab32 knockdown on mitochondria.** (A) Representative [transmission electron microscope](https://www.baidu.com/s?tn=98157817_hao_pg&usm=2&wd=transmission%20electron%20microscope%E7%BF%BB%E8%AF%91&ie=utf-8&rsv_pq=a712b792002fe85a&oq=%E9%80%8F%E5%B0%84%E7%94%B5%E9%95%9C%E8%8B%B1%E8%AF%AD&rsv_t=4442boxupT4C5j5XyOI%2F9OqMZYxaiaVvRuz3VrL38%2BgNeImgWyW4TOgQE8TyxCdHmtagE7AM&sa=re_fy_huisou) image showing mitochondria morphological changes accompanying Schwann cell pyroptosis induced by LPS/ATP. Scale bar = 2 µm. (B) Quantitative data of the average number of mitochondria per unit area. (C) Quantitative data of the average volume of mitochondria per unit area. ***p* < 0.01 compared to the LA/shRab32 group. **p* < 0.05 compared to the LA/shRab32 group.


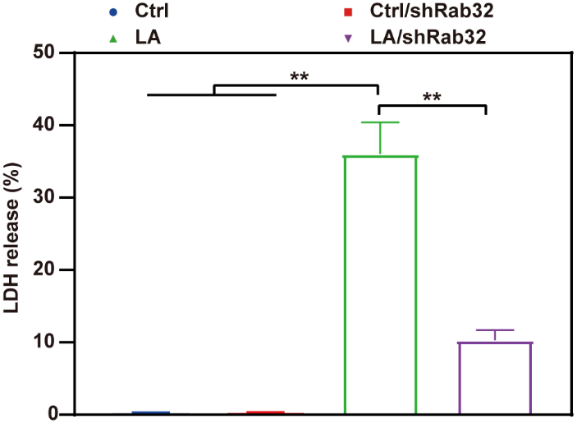


**Figure S2. Quantification of LDH release indicating Schwann cell damage.** ***p* < 0.01 compared to the LA group. **p* < 0.05 compared to the LA group.


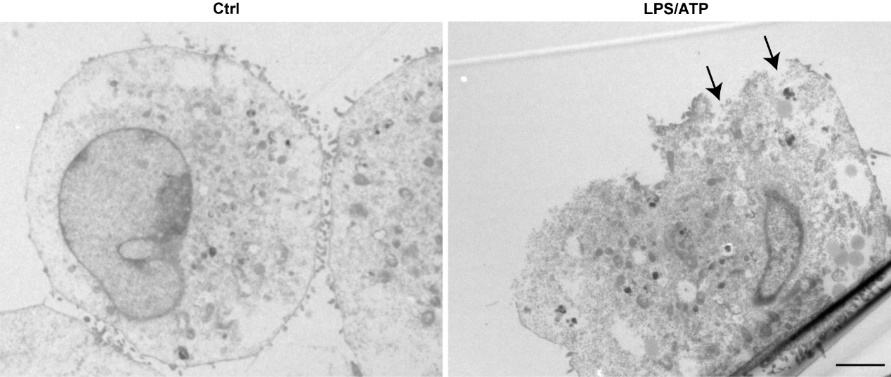


**Figure S3. Representative** [**transmission electron microscope**](https://www.baidu.com/s?tn=98157817_hao_pg&usm=2&wd=transmission%20electron%20microscope%E7%BF%BB%E8%AF%91&ie=utf-8&rsv_pq=a712b792002fe85a&oq=%E9%80%8F%E5%B0%84%E7%94%B5%E9%95%9C%E8%8B%B1%E8%AF%AD&rsv_t=4442boxupT4C5j5XyOI%2F9OqMZYxaiaVvRuz3VrL38%2BgNeImgWyW4TOgQE8TyxCdHmtagE7AM&sa=re_fy_huisou) **image showing morphological changes accompanying Schwann cell pyroptosis induced by LPS/ATP.** The black arrows represent the pores on the cell membrane, which is also one of the characteristic signs of pyroptosis. Scale bar = 2 µm.


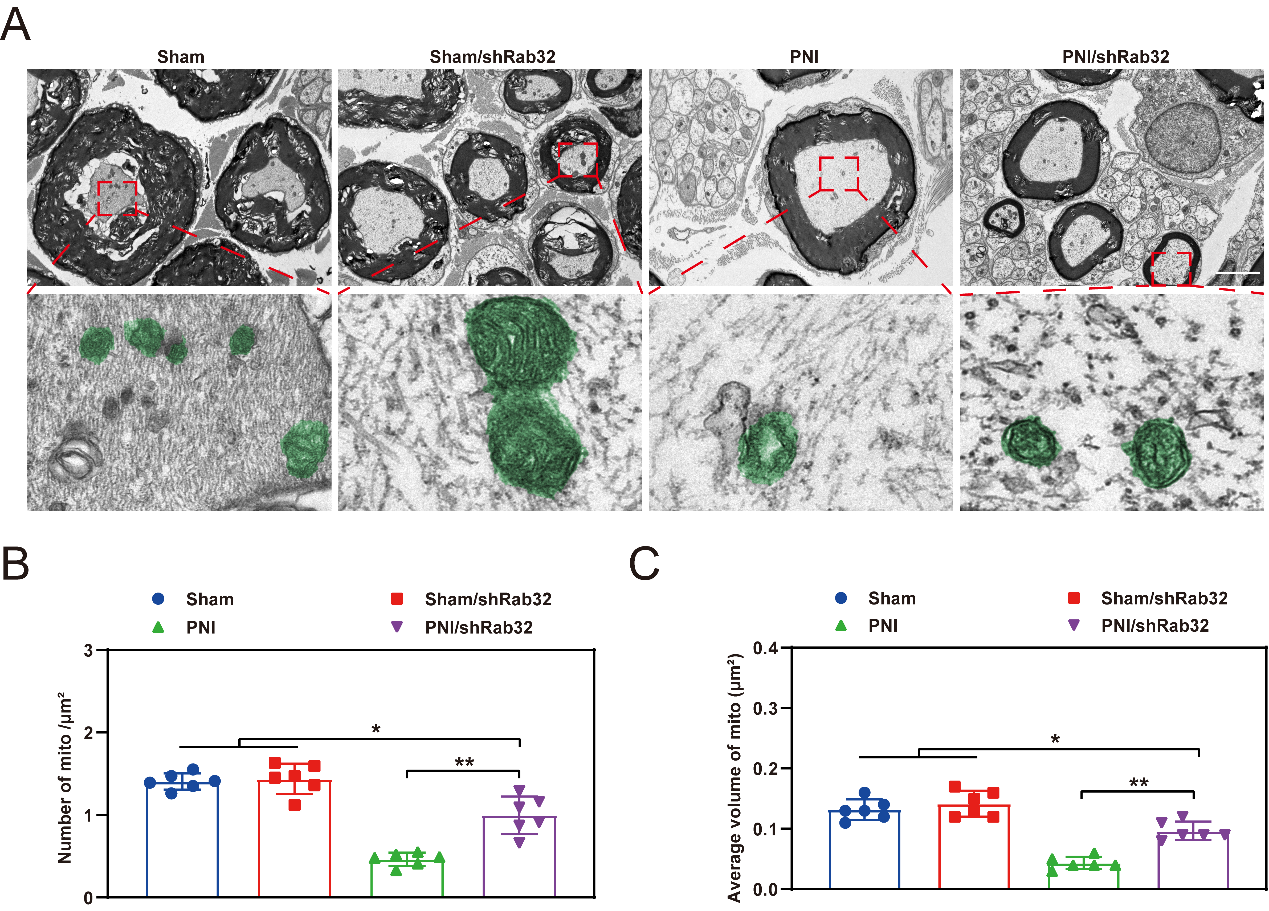


**Figure S4. The impact of Rab32 on mitochondrial morphology in Schwann cells following peripheral nerve injury.** (A) Representative [transmission electron microscope](https://www.baidu.com/s?tn=98157817_hao_pg&usm=2&wd=transmission%20electron%20microscope%E7%BF%BB%E8%AF%91&ie=utf-8&rsv_pq=a712b792002fe85a&oq=%E9%80%8F%E5%B0%84%E7%94%B5%E9%95%9C%E8%8B%B1%E8%AF%AD&rsv_t=4442boxupT4C5j5XyOI%2F9OqMZYxaiaVvRuz3VrL38%2BgNeImgWyW4TOgQE8TyxCdHmtagE7AM&sa=re_fy_huisou) image showing mitochondrial morphological changes accompanying Schwann cell pyroptosis induced by PNI. Scale bar = 2 µm. (B) Quantitative data of the average number of mitochondria per unit area. (C) Quantitative data of the average volume of mitochondria per unit area. ***p* < 0.01 compared to the PNI/shRab32 group. **p* < 0.05 compared to the PNI/shRab32 group.


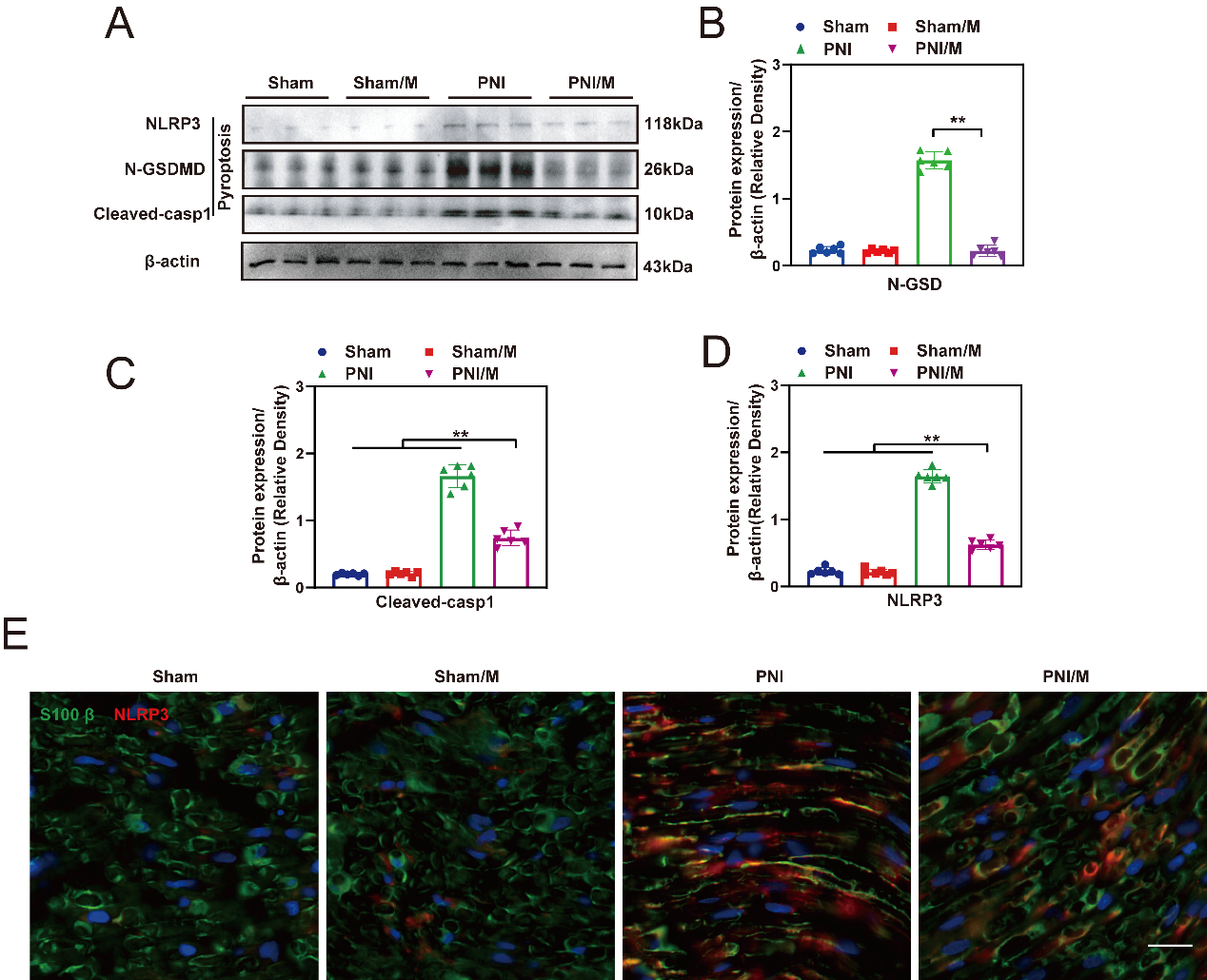


**Figure S5. Evaluation of the effect of MitoQ on pyroptosis in peripheral nerve injury.** (A) Western blotting analysis was conducted to evaluate the levels of pyroptosis-associated proteins in nerve tissues. β-actin was used as an internal control. (B-D) Levels of pyroptosis-associated proteins were quantified based on semi-quantitative band analysis. (E) Representative immunofluorescence images displaying NLRP3 (red) and S100β (green) in nerve tissues. ***p* < 0.01 compared to the PNI/M group. **p* < 0.05 compared to the PNI/M group. Scale bar = 20 µm.
